# Body Composition Analysis in Young Patients with Recent Diagnosis of Multiple Sclerosis: An Exploratory Study

**DOI:** 10.3390/jcm15135241

**Published:** 2026-07-04

**Authors:** Riccardo Orlandi, Sara Bendazzoli, Francesca Gobbin, Alessandra Carcereri de Prati, Elena Butturini, Sofia Mariotto, Valentina Cavedon, Chiara Milanese, Alberto Gajofatto

**Affiliations:** Department of Neurosciences, Biomedicine and Movement Sciences, University of Verona, 37134 Verona, Italy; riccardo.orlandi@aovr.veneto.it (R.O.); sara.bendazzoli@studenti.univr.it (S.B.); francescagobbin@gmail.com (F.G.); alessandra.carcererideprati@univr.it (A.C.d.P.); elena.butturini@univr.it (E.B.); sofia.mariotto@univr.it (S.M.); valentina.cavedon@univr.it (V.C.); chiara.milanese@univr.it (C.M.)

**Keywords:** multiple sclerosis, BMI, sarcopenia, brain volumes, oxidative stress

## Abstract

**Background/Objectives**: The relationship between body composition (BC), sarcopenia, and multiple sclerosis (MS) remains poorly understood. A high body mass index (BMI) is associated with a higher risk of MS and brain atrophy. However, limited data are available on BC in patients in the early stages of the disease. This study investigates differences in BC and sarcopenia between early-diagnosed patients with MS (pwMS) and healthy controls (HC), while exploring correlations with brain atrophy and biomarkers of oxidative stress and axonal injury. **Methods**: This project is part of BPS-ARMS, a cross-sectional study conducted in 2019–2022 at Verona University involving 51 participants aged 18–40 years, diagnosed with MS in the last two years, and currently not taking disease-modifying drugs. Seventeen (69% females) pwMS consented to be enrolled in this sub-study, matched by age and body mass index (BMI) to 17 HC; BC was assessed using Dual-Energy X-ray Absorptiometry (DXA). Collected variables included BMI, fat and lean mass, and sarcopenia index (SI). A brain MRI scan was performed in pwMS between 6 months before and 1 month after inclusion, to assess T2 lesion, normalized brain (NBV), white matter (WMV) and gray matter (GMV) volumes, and presence of gadolinium-enhancing (Gd+) lesions. Biomarker analysis was performed on blood samples collected at baseline. Oxidative stress was assessed as plasma gluthatione (GSH) and gluthatione disulphide (GSSG) levels and STAT1 phosphorylation at Tyr 701 (pSTAT) in peripheral blood cells, while axonal damage was measured as serum neurofilament light chain (NfL) levels. **Results**: A significantly lower SI was found in pwMS compared to HC (*p* = 0.038), particularly in female cases. In the pwMS group, WMV was inversely correlated with SI (*p* = 0.028) and lean body mass (*p* = 0.016). BMI was inversely correlated with WMV (r = −0.658, *p* = 0.02). A significant inverse correlation of plasma GSSG level was found with SI (r = −0.546, *p* = 0.023) and lean mass (r = −0.585, *p* = 0.014); the ratio of GSH over GSSG (GSH/GSSG) was directly correlated with SI (r = 0.518, *p* = 0.036) and lean mass (r = 0.568, *p* = 0.017). **Conclusions**: Patients with early-stage untreated MS and low-grade disability are more prone to sarcopenia than HC. Moreover, MS subjects with higher BMI show lower brain white matter volume and a lower global brain volume.

## 1. Introduction

Multiple sclerosis (MS) is a chronic inflammatory disease of the central nervous system (CNS) potentially leading to neurological disability in young adults. Although MS etiology is unknown, immunopathophysiology stands on a complex interplay between environment and genetic predisposition; several acquired risk factors have been called into questions including, among others, lifestyle and diet [1].

A high body mass index (BMI)—particularly in the range corresponding to obesity, in women and children—has been associated with a higher risk of MS and a higher risk of disability progression [2,3,4]. Moreover, higher BMI is associated with lower brain volumes in MS patients [5]. However, BMI does not distinguish fat mass from lean mass and therefore does not provide a direct measure of actual body composition as Dual-Energy X-ray Absorptiometry (DXA) can do. Using this method, it has been observed that men with longstanding MS have significantly less lean body mass and more fat mass than healthy controls (HCs) with the same BMI [6]. In addition, it has been reported that MS patients with high disability have a significant decrease in lean mass and an increase in fat mass, regardless of disease duration [7].

Currently, it remains unclear whether body composition (BC) changes observed in patients with MS compared to controls are either indirect consequences of the disease’s impact on lifestyle habits (e.g., physical activity and diet) or intrinsic features of subjects with MS potentially related to disease pathogenic mechanisms or a combination of both. Previous studies on BC in MS lacked homogeneity in patient selection in terms of disease duration and severity. Moreover, no studies have currently investigated the relationship between BC and brain atrophy in MS.

A role of oxidative stress (OS) in MS pathogenesis has been proposed, as indicated by the biochemical analysis of cerebrospinal fluid and blood samples and animal models of MS. Glutathione (GSH) is the main antioxidant in the brain and plays a key role in the detoxification of reactive species. An important phenomenon in MS is an altered glutathione homeostasis [8]. The interplay between obesity and the development of a pro-inflammatory state mediated by OS is known. Obesity triggers chronic inflammation, alteration of the blood–brain barrier and brain metabolism, production of ROS, and generation of OS [9]. However, the relationship between BC and antioxidant levels in patients with MS is still unknown.

Hence, the aims of the present research are to examine the following measures in a cohort of young patients with recent MS diagnosis:

- The differences in total and regional body composition measured by DXA between HCs and MS cases matched by sex, age, and BMI.
- The differences in total and regional body composition according to demographic variables, clinical data, OS biomarkers, and magnetic resonance imaging (MRI) measures.

## 2. Materials and Methods

### 2.1. BPS-ARMS Project

The present study is part of the project Biopsychosocial model of resilience in young adults with multiple sclerosis (BPS-ARMS), a cross-sectional study carried out at the University of Verona between February 2019 and February 2022, with the scope of assessing a panel of prespecified biological, psychological, and social characteristics of a group of young patients recently diagnosed with MS [10]. People with MS (pwMS) were enrolled in the BPS-ARMS project at the Regional Centre for Multiple Sclerosis of the University Hospital of Verona according to the following criteria:

- Inclusion Criteria
  1. Age range 18–45 years;
  2. Diagnosis of MS in the two years prior to study inclusion, according to 2017 revision of McDonald Criteria [11];
  3. Brain MRI performed within one month of enrolment according to the protocol listed below (or in the six months prior to enrolment for clinical purposes with the same acquisition protocol);
  4. Knowledge of the Italian language;
  5. Signed written informed consent.
- Exclusion Criteria
  1. Clinically relevant cognitive deficits;
  2. Treatment with any disease-modifying therapy for MS at the time of inclusion and completion of study procedures (i.e., one month);
  3. Administration of steroids less than thirty days prior to inclusion.

After signing the informed consent form, patients underwent an enrolment visit, including demographic characteristic collection and clinical evaluation encompassing medical history, date of initial MS symptoms, type of onset (relapsing–remitting—RRMS, or primary progressive—PPMS). In addition, study patients underwent a neurological examination for expanded disability status scale (EDSS) scoring [12].

Within one month from signing the informed consent form, patients underwent all experimental procedures of the various work packages included in the BPS-ARMS protocol (with the exception of an MRI scan, which could have been performed up to six months prior to enrolment for clinical purposes—see inclusion criteria listed above). For the present study, the following variables were specifically collected and analyzed: brain MRI measures, body composition measured by DXA, and biomarkers of oxidative stress and axonal injury (i.e., neurofilament light chain) assessed in blood samples. All procedures listed above were completed to ensure timely access to appropriate MS therapy for each patient. Patients were asked to be in a fasted state at blood collection; however, they had no specific restrictions regarding physical exercise the day before study procedures. Experimental study procedures did not influence subsequent treatment decisions.

### 2.2. MRI Protocol

For each participant, a brain MRI carried out in the six months preceding the signing of the informed consent, or within one month of the same, was acquired with a 1.5 Tesla scanner (Philips Ingenia^®^ or Signa Artist^®^, Amsterdam, The Netherlands) according to a standardized protocol, which included pre- and post-contrast 3D T1-weighted, 3D Fluid-Attenuated Inversion Recovery (FLAIR) and 3D Double Inversion Recovery (DIR) volumetric sequences.

The number of contrast-enhancing lesions was determined on T1-weighted sequences after intravenous injection of gadolinium-based contrast.

Measurement of global brain, gray matter, and white matter volumes was performed using SIENAX (Structural Image Evaluation, using Normalization, of Atrophy—Cross-Sectional) software (https://web.mit.edu/fsl_v5.0.10/fsl/doc/wiki/SIENA(2f)UserGuide.html, accessed on 31 August 2025), which can estimate brain volumes from a single image, normalized for skull size using T1-weighted scans [13].

For the analysis of the volume of brain lesions on T2-weighted MRI sequences, the open source software ITK-SNAP version 4.0 (https://www.itksnap.org/pmwiki/pmwiki.php, accessed on 31 August 2025) was used for semi-automatic segmentation of lesions [14].

### 2.3. DXA

Body composition was assessed through DXA with a QDR Explorer fan beam densitometer (Hologic, Bedford, MA, USA) available in the Sports Sciences section of the Department of Neuroscience of Verona University. Quality control of the DXA scanner is performed daily in the laboratory, prior to actual use, using a Hologic encapsulated spine phantom (Hologic Inc., Bedford, MA, USA) supplied by the manufacturer to avoid possible drift of baseline references. During the investigations for the project, the Hologic software version 12.6 was calibrated daily, and the phantom measurements used (bone mineral density, bone mineral content, and area values) were within the upper and lower limits of the ranges used, i.e., from the baseline, +/− 1.5%. In addition, the long-term performance of the DXA scanner was monitored throughout the study period, during which the measurements of the phantom used for calibration remained stable, with a coefficient of variation for each of the measurements of less than 1%.

All participants were asked not to engage in strenuous physical activity the day before the measurement and were also required not to engage in any physical exercise on the morning of the measurements. Before the scan, participants were asked to empty their bladder and remove all metal objects. Relative body mass (weight) data were taken to the nearest 0.1 kg using an electronic scale (Tanita electronic scale BWB-800 MA, Tokyo, Japan), and height was measured with a Harpenden stadiometer (Holtain Ltd., Crymych, Pembrokeshire, UK) to the nearest mm; during these measurements, participants wore only underwear.

Participants underwent three scans in the following order: whole body, proximal femur, and lumbar spine in the postero-anterior projection. All scans were performed according to standard protocols. Specifically, whole-body scans were performed according to “The Best Practice Protocol for the assessment of whole-body composition by DXA” [15], which describes recommendations for positioning subjects on the densitometer table. All scans were analyzed using Hologic Discovery software for Windows XP version 12.6.1. In the whole-body scan, the technician locates specific anatomical landmarks to differentiate the standard regions of interest (arms and legs and trunk). In addition to the standard analysis of regional body composition, the technician manually defines additional regions of interest (the android region) to more precisely localize the central fat distribution in the subject. The android region is defined as the area between the ribs and the pelvis [16]. The automatic analysis mode is used by default for the proximal femur, lumbar spine and forearm scans, which are subsequently checked and corrected by the technician. All DXA scans were reviewed and analyzed by the same research engineer.

Body composition variables of interest derived from the whole-body scan include fat-free soft tissue mass (FFSTM, kg) or “lean mass”, fat mass (FM, kg), and sarcopenia index (SI, kg/m^2^). The values are calculated at the level of the whole body and at the regional level (arms, legs and trunk).

SI is calculated by dividing fat-free appendicular lean mass (kg) by height squared (m^2^). The International Working Group on Sarcopenia considers an SI value of less than 7.23 kg/m^2^ for males and less than 5.67 kg/m^2^ for females to be the cut-off for sarcopenia [17].

For comparison, body composition data from retrospectively collected HCs matched 1:1 to MS cases by sex, age and BMI were included in the analysis. HC data were taken from the repository of the Sports Sciences section of the Department of Neuroscience of Verona University and were obtained with the same equipment and protocol described above.

### 2.4. Serum Biomarkers

Within one month of completion of screening, patients included in the BPS-ARMS study underwent blood sampling from a peripheral vein for biomarker analysis.

Neurofilament light chain (NfL) serum concentration was assessed at Centro Piattaforme Tecnologiche of Verona University. Serum was obtained from tubes without any additives. The serum samples were allowed to clot for 30 min at room temperature; they were then centrifuged, aliquoted at room temperature, and stored at −80 °C. The freezer temperature was continuously monitored, and samples were thawed only before analysis. The NfL concentration was measured in duplicate in all available serum samples. The analysis was conducted using the same batch of reagents by investigators blinded to clinical data, employing the Single Molecule Array (SIMOA) Nf-light^®^ kit on the SR-X immunoassay analyzer, Simoa™ (Quanterix Corp, Boston, MA, USA), which utilizes ultrasensitive paramagnetic bead-based enzyme-linked immunosorbent assays. Briefly, frozen samples and calibrators were equilibrated to room temperature and diluted with a specific sample diluent. Calibrators, samples, detector, and beads were dispensed into each well, and plates were incubated at 30 °C with shaking at 800 rpm for 30 min. Following washing steps, 100 μL of streptavidin-*β*-galactosidase (SBG) was added to each well, and plates were incubated again at 30 °C with shaking at 800 rpm for 10 min. After further washing steps, beads were resuspended twice at 1000 rpm for 1 min. A final washing step was performed, and the plates were dried for 10 min before being transferred to the SR-X for reading.

Oxidative stress was assessed in blood plasma by measuring the concentration of GSH and GSSG, as described by Giustarini et al. [18]. Briefly venous blood samples (5 mL) from MS patients and HC were collected into standard sterile polystyrene vacuum tubes containing K_3_EDTA, in the presence of 20 mM N-ethylmaleimide (NEM), a thiol-alkylating agent used to prevent the artificial auto-oxidation of GSH during sample handling. Samples were immediately centrifugated at 15,000× g for 3 min, and the obtained plasma was deproteinized with 5% trichloroacetic acid (TCA). After centrifugation to remove precipitated proteins, the clear supernatant was divided into two aliquots for separate determination of GSH and GSSG.

GSH concentration was quantified by measuring GS-NEM conjugate levels using reverse-phase high-performance liquid chromatography (HPLC) (JASCO, Cremella, LC, Italy) on a C18 column (4.6 × 150 mm, 5 μm). Chromatographic detection was performed by recording the signals at 265 nm. GS-NEM was eluted under isocratic condition using 94% phase A (0.25% acetic acid in water) and 6% phase B (100% acetonitrile) with a flow rate of 1.25 mL/min.

GSSG levels were determined using a spectrophotometric GSH recycling assay on TCA-treated samples, as previously described [18]. As residual NEM may interfere with the enzymatic reaction, the excess of NEM was removed from the supernatant by extraction with 10 vol of dichloromethane. After vigorous mixing and centrifugation to allow phase separation, the upper aqueous phase was collected for analysis. In the recycling assay, GSSG was reduced to GSH by glutathione reductase in the presence of NADPH. The generated GSH reacts with 5,5′-dithiobis(2-nitrobenzoic acid) to form the yellow chromophore 5-thio-2-nitrobenzoic acid, whose formation was monitored spectrophotometrically at 412 nm.

Activation of the STAT1 pathway (pSTAT1) was assessed in peripheral blood mononuclear cells (PBMCs) through Western blot analysis using anti-phospho-Tyr701 STAT1 and anti-STAT1 antibodies [19].

PBMCs were isolated from heparinized blood through density-gradient centrifugation using Lymphoprep Ficoll-Isopaque (Axis-Shield, Oslo, Norway) according to the manufacturer’s instructions. The cells were lysed on ice for 15 min in 20 mM HEPES buffer (pH 7.4) containing 420 mM NaCl, 1% Nonidet P40, 1 mM EGTA, 1 mM EDTA and protease and phosphatase inhibitor cocktails. Lysates were centrifuged at 12,000× g for 15 min at 4 °C, and supernatants were collected for protein analysis. Equal amounts of proteins (50 μg/lane) were separated on 7.5% SDS-polyacrylamide gels and electroblotted onto a PVDF membrane (Immobilon P, Millipore, Bedford, MA, USA). Membranes were incubated with anti-phosphoSTAT1 (Tyr701) primary antibody (Cell Signaling Technology, Danvers, MA, USA), and, after washing, with anti-rabbit IgG-peroxidase conjugate (Amersham Biosciences, Little Chalfont, Buckinghamshire, UK). The immunoreactive proteins were detected using an enhanced chemiluminescence detection system (WBKLS0500, Millipore). Membranes were then stripped and re-probed with anti-STAT1 antibody to determine total STAT1 levels. Band intensities were quantified through densitometric analysis using the ChemiDoc XRS Imaging System (Bio-Rad, Hercules, CA, USA) and were quantified using ImageLab 6.1.0 (BioRad). STAT1 activation was expressed as the ratio of phosphorylated STAT1 (pSTAT1) to total STAT1.

### 2.5. Statistical Analysis

Demographic (age, sex), clinical (EDSS, DXA) and MRI variables are described in terms of mean and standard deviation (SD) or median and range, as appropriate, for quantitative variables, and in terms of occurrence (*n*) and percentage frequency (%) for nominal data. The statistical analysis was carried out using the “Jamovi” software version 2.6 [20]. Comparisons between the two groups (MS and controls) and between MS subjects divided by sex, age, EDSS and SI were carried out using the T-test for variables with continuous/normal distribution or Mann–Whitney test for variables with non-normal distribution. For the statistical analyses carried out within the MS sample, the Pearson correlation coefficient was used for variables with continuous distribution and the Spearman correlation coefficient for nonparametric variables. Although the number of variables was high in comparison to the sample size, no formal adjustment of the analysis was performed given the exploratory design of the study.

A *p*-value < 0.05 was considered significant for a two-tailed test.

## 3. Results

### 3.1. Sample Description and Body Composition Meausers in pwMS and HC

From February 2019 to February 2022, 51 eligible pwMS (33 female, mean age 35.1 years) were consecutively enrolled in the BPS-ARMS study.

From the starting sample, 17 pwMS (10 females) agreed to proceed with body composition analysis using DEXA. Descriptive and body composition variables are reported in Table 1.

At enrollment, the mean age of the sample was 35.1 years ± 6.26. The median interval between MS diagnosis and enrolment was 6.5 months (range 0–23). The median interval elapsed between disease onset and enrolment was 18 months (range 1–115). At recruitment 14 patients had an RR and three had a PP clinical course. The median EDSS score was 1, with a range from 0 to 4. A contrast-enhanced brain MRI was available for 14 of the 17 subjects enrolled in the study; of these patients, four (28.6%) had one or more enhancing lesions.

At enrolment, 4 out of 17 pwMS (all females, 23.5%) had an SI below the cut-off established by the International Group on Sarcopenia, compared to one female HC (5.9%). Of note, none of pwMS and HCs participated in sports at a competitive level.

Comparing body composition variables between MS patients and HCs, there was a statistically significant difference in SI between the two groups (*p* = 0.038). After stratifying by sex, the mean SI was confirmed to be significantly lower in MS cases than in controls for both females and males (6.03 ± 0.61 vs. 6.81 ± 1.02, *p* = 0.043 in females; 7.96 ± 0.43 vs. 8.65 ± 0.62, *p* = 0.038 in males).

In pwMS, women showed a significantly lower median lean body mass (38.37 kg, range 34.38–50.73) compared to men (54.26 kg, range 47.84–62.72, *p* < 0.001), as well as a lower median SI (5.86 kg/m^2^, range 5.43–7.41) compared to men (7.92 kg/m^2^, range 7.39–8.78, *p* < 0.001). These statistically significant differences were comparable to the results obtained in controls.

No statistically significant differences in body composition measures were observed when stratifying patients by age (cut-off, 35 years), disease duration (cut-off, 18 months), and EDSS score (cut-off, 2).

### 3.2. Association of Body Composition and MRI Measures in pwMS

No statistically significant differences were observed for body composition-related variables when comparing pwMS who were found to have Gd+ lesions at brain MRI scans and participants without such lesions.

**Figure 1:**
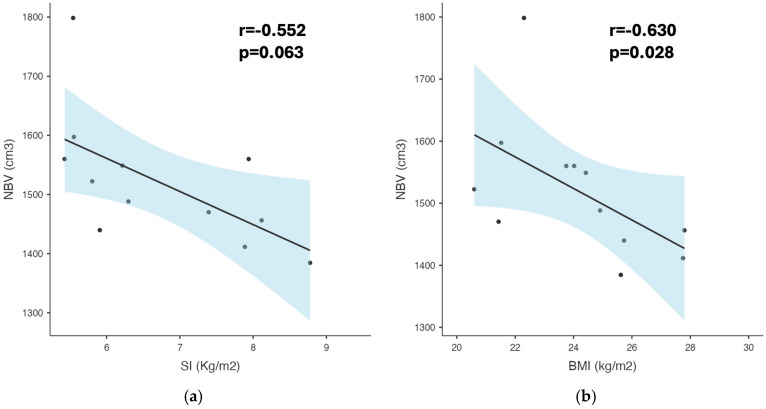
Association between NBV and body composition measures in pwMS. (**a**) Correlation between SI and NBV; (**b**) correlation between BMI and NBV. Circles represent individual case values, the black line represents the regression line, while the blue area represents the confidence interval of the correlation coefficient estimate.

**Figure 2:**
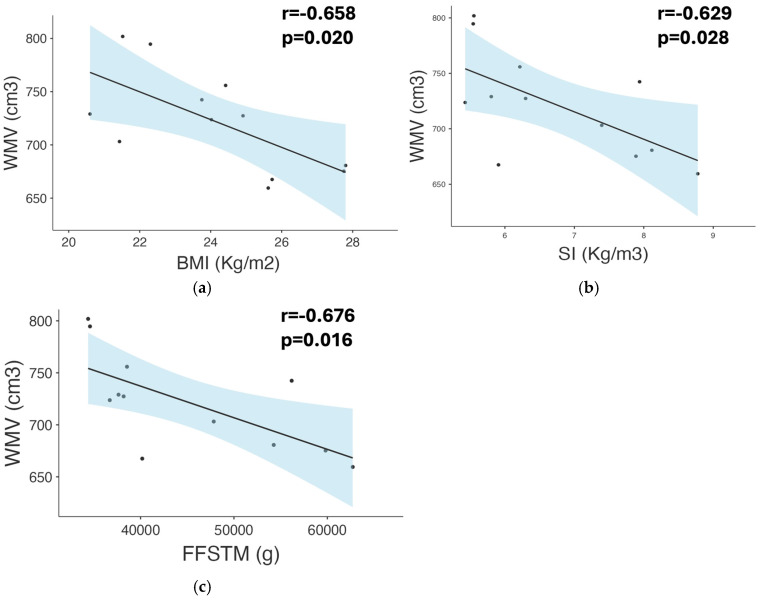
Association of WMV with body composition measures. (**a**) Inverse correlation between WMV and NBV; (**b**) inverse correlation between WMV and SI; (**c**) inverse correlation between WMV and FFSTM. Circles represent individual case values, the black line represents the regression line, while the blue area represents the confidence interval of the correlation coefficient estimate.

NBV showed a trend towards a significant inverse correlation with SI (r = −0.552, *p* = 0.063, Figure 1a), while it was inversely correlated with BMI (r = −0.630, *p* = 0.028, Figure 1b). WMV was inversely correlated with BMI (r = −0.658, *p* = 0.020, Figure 2a), SI (r = −0.629, *p* = 0.028, Figure 2b) and FFSTM (r = −0.676, *p* = 0.016, Figure 2c).

No significant differences were found in median NBV, GMV and WMV when pwMS were stratified according to the samples’ median value of FFSTM (cut-off, 41.71 kg) and of SI (cut-off, 6.57) as shown in Supplementary Material.

### 3.3. Correlation Analysis Between Serum Biomarkers and Body Composition Variables

No significant correlation was observed between serum NFL concentration and any of the body composition measures. Moreover, no significant differences in body composition measures were observed between patients with low or high expression of pSTAT1 (Supplementary Material).

**Figure 3:**
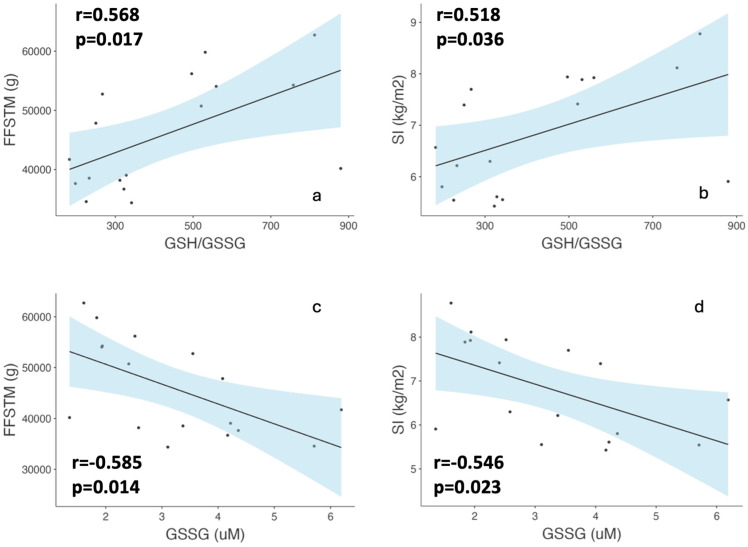
Correlation between body composition measures and oxidative stress. (**a**) Direct correlation between GSH/GSSG ratio and FFSTM; (**b**) direct correlation between GSH/GSSG ratio and SI; (**c**) inverse correlation between GSSG and FFSTM; (**d**) inverse correlation between GSSG and SI. Circles represent individual case values, the black line represents the regression line, while the blue area represents the confidence interval of the correlation coefficient estimate.

The GSH/GSSG ratio was found to have a statistically significant direct correlation with FFSTM (r = 0.568, *p* = 0.017, Figure 3a) and SI (r = 0.518, *p* = 0.036, Figure 3b). GSSG was found to have a statistically significant inverse correlation with FFSTM (r = −0.585, *p* = 0.014, Figure 3c) and SI (r = −0.546, *p* = 0.023, Figure 3d).

## 4. Discussion

### 4.1. Relationship Between Sarcopenia and Multiple Sclerosis

In the present study, DXA-based body composition analysis revealed that young pwMS exhibit a significantly lower appendicular SI compared to HCs matched for sex, age, and BMI. While other anthropometric measures were comparable between groups, these findings suggest that MS patients may be more predisposed to sarcopenia than the general population. Notably, this increased susceptibility is evident even in patients under the age of 46, with an EDSS score below 4, and within two years of diagnosis.

To our knowledge, this is the first study in the field that includes patients with an early diagnosis of MS and a low disability level, thus providing a more homogenous cohort compared to previous works [21].

In addition, the patients included in our study were not being treated with DMDs and steroids that could have influenced the examined variables.

Wingo et al. previously observed that men with MS had significantly less lean body mass and more fat mass than age- and BMI-matched HCs as measured by DXA, particularly in the arms [6]. However, MS cases had a mean age of 44 years, EDSS score of 4 and disease duration of 12 years, which were higher compared to our sample, possibly influencing body composition measures.

In fact, a different study found that MS females with EDSS scores of 4.5–6.5 had significantly lower lean mass and higher fat mass percentages compared to MS females with EDSS scores of 1.0–4.0, independent of disease duration [7]. However, age was significantly higher in the more disabled patient group, and body composition measures were acquired using bioelectrical impedance analysis rather than DXA, which is the gold standard for body composition assessment used in our study.

In our sample, four patients (all women) out of 17 total cases had an SI value lower than the cut-off for sarcopenia diagnosis according to international guidelines.

Sarcopenia is a syndrome characterized by a progressive and generalized loss of skeletal muscle mass and strength associated with aging, female sex, sedentary lifestyle, malnutrition, being underweight, and chronic medical conditions [22,23,24].

Patients suffering from MS with reduced mobility are clearly predisposed to secondary sarcopenia. However, little is known about the possible association between the MS diagnosis and body composition abnormalities independent of motor impairment. Yuksel et al. examined the prevalence of sarcopenia among 101 patients with MS who were able to ambulate without assistance (like the sample in our study) and found that 19% of patients were sarcopenic using a combination of ultrasound and strength tests of appendicular muscles; this proportion dropped to 11% using whole-body bioelectrical impedance analysis, with similar findings in both sexes [25].

Although pathophysiological processes that can lead to sarcopenia in MS are not established, they could involve muscle denervation, mitochondrial dysfunction, oxidative stress at the cellular level, circulating inflammatory cytokines and changes in hormone levels [26,27,28]. In our study, we found an association between blood biomarkers of oxidative stress included in the research protocol, sarcopenia, and lean body mass. In particular, we found a significant inverse correlation of plasma GSSG levels with SI and lean body mass. On the contrary, the GSH/GSSG ratio was directly correlated with SI and lean body mass. MS patients with higher blood levels of oxidative stress biomarkers therefore have a greater tendency towards sarcopenia. Oxidative stress is a factor correlated with a higher prevalence of sarcopenia: sarcopenic subjects older than 65 years have higher blood levels of GSSG and lower levels of GSH compared to non-sarcopenic controls [29]. A similar result to the one found in our study was reported in a study that evaluated the levels of GSH, GSSG, and their ratio in women with polycystic ovary syndrome: it was noted how lean body mass has a direct correlation with GSH/GSSG and an inverse correlation with GSSG [30]. Disease-modifying drugs (DMDs) can also have an impact on the risk of sarcopenia in patients with MS; in particular, the risk seems to be higher in obese patients [31]. This increased risk of sarcopenia related to treatment does not apply to our sample, as included patients were DMD-naïve and had not taken steroids in the 30 days prior to enrolment.

### 4.2. Relationship Between BMI and Brain Volume

Our study shows an inverse correlation between BMI and brain volumes in patients with MS: the higher the BMI, the lower the brain white matter volume and global brain volume. To the best of our knowledge, our study is the only one to have analyzed the association of BMI with brain volumes in young patients newly diagnosed with MS and not exposed to DMDs. This result has been found in other studies that have evaluated the correlation between brain volumes and BMI in populations other than MS or in MS participants with significantly different characteristics compared to our sample.

Boyle et al. evaluated 963 subjects with Alzheimer’s disease by studying the correlations between physical activity, BMI and brain atrophy: it was found that physical activity was independently associated with a higher global and regional brain volume and reduced ventricular dilation. Patients with a higher BMI had lower global and regional brain volumes [32]. A population-based, multicenter cohort study carried out in 2024 evaluated 1074 HCs between the ages of 25 and 83 and found that a higher BMI was associated with a lower brain volume, a higher volume of white matter lesions, and abnormal microstructural integrity [33]. A different result was found in a study evaluating 3098 school-age children with neuroimaging: compared to children with a normal weight, those who were underweight had a lower total brain and white matter volume [34].

Patients with a clinically isolated syndrome and BMI 30 kg/m^2^ or more from the BENEFIT trial were at a higher risk of developing MS and had an increased relapse rate; however, a larger reduction in brain volume on MRI was found only in obese smokers compared to normal weight smokers [35].

A recent study evaluated 13 women with MS, with a mean age of 44 years, mean BMI of 26.62 kg/m^2^ and mean time from disease onset of 13.8 years. A strong negative correlation was found between BMI and global brain volume, suggesting an association between increased BMI and decreased brain volume [5].

The results found in our study may be relevant in consideration of the data from a recent longitudinal study showing that obesity, in a large group of 1066 MS patients with recent diagnosis and a mean age of 33 years, was associated with greater disease severity and worse outcomes. It was noted that the risk of achieving EDSS = 3 over 6 years increased significantly in patients with BMI ≥ 30 kg/m^2^ compared to patients with BMI < 30 kg/m^2^ [3]. In addition, Wu et al. reported that obese patients from the Swedish MS registry were at a higher risk of faster disease progression, poorer health-related quality of life, and more rapid cognitive decline [4].

### 4.3. Relationship Between Brain Volumes, Lean Mass and Sarcopenia Index

Our study shows that cerebral white matter volume is inversely related to sarcopenia index and lean body mass: it seems that as muscle mass and the sarcopenia index increase, brain volume decreases. This finding appears counterintuitive and has no counterpart in other studies.

For example, a study of 140 patients with Alzheimer’s disease found that lower lean body mass and the presence of sarcopenia were associated with brain atrophy and worse cognitive performance [36].

It could be hypothesized that sarcopenic MS patients may have a more active disease with more inflamed and oedematous brain parenchyma, resulting in increased white matter volume on brain MRI compared to non-sarcopenic patients.

The small sample size of our study prevents subgroup analysis to confirm this hypothesis, suggesting that the correlation observed between sarcopenia and white matter brain volume in MS cases needs to be interpreted with caution. A study on a larger sample and with a longitudinal design could be useful for evaluating this data.

### 4.4. Limitations of the Study

The main limitation of our study is the low number of patients in the sample. In addition, there is no specific information on the lifestyles of patients and HCs, including on diet, levels of physical activity and smoking habits.

## 5. Conclusions

Early-diagnosed untreated MS patients with low disability are more prone to sarcopenia than healthy controls even before significant mobility reduction occurs. MS patients with higher blood levels of oxidative stress biomarkers have a greater tendency towards sarcopenia. In addition, MS cases with higher BMI show more brain atrophy.

If replicated in larger cohorts, the results of this study could have relevant clinical implications. First, beginning at the time of diagnosis, young patients with MS should be encouraged to regularly adopt lifestyle habits that may prevent sarcopenia, starting with adequate exercise. Second, patients should be provided with nutritional counseling aimed at preventing or improving sarcopenia and maintaining optimal BMI. Third, patients should be referred early to rehabilitation in order to slow down sarcopenia occurrence. All these strategies could provide effects that counteract the mechanisms leading to sarcopenia, which could be associated with both MS pathophysiological processes and reduced physical activity.

## Figures and Tables

**Table 1:** Clinical, demographic and body composition variables of pwMS and HCs.

	MS Cases (*n* = 17)		HC (*n* = 17)		*p*
Sex—*n* (%) Females Males	10 (41.2) 7 (58.8)		10 (41.2) 7 (58.8)		>0.99
Age at enrolment—mean ± SD Females Males	35.1 ± 6.26 33.6 ± 6.73 36.1 ± 6.01		36.4 ± 6.87 34.3 ± 6.90 37.9 ± 6.81		0.553
EDSS—median (range)	1.0 (0–4.0)		NA		
Time from diagnosis at enrolment, months—median (range)	6.5 (0–23)		NA		
MS disease course—n (%) Relapsing–remitting Primary progressive	14 (82.4) 3 (17.6)		NA		
BMI—median (range) Females Males	24.4 (19.3–36.1) 24.2 (19.3–36.1) 25.6 (21.4–27.8)	*p* = 0.669 *	24.3 (19.2–36.2) 23.9 (19.2–36.2) 24.3 (20.4–28.0)	*p* = 0.813 *	0.866
FM (kg)—median (range) Females Males	19.87 (10.42–44.84) 20.65 (13.53–44.83) 17.27 (10.42–27.34)	*p* = 0.230 *	17.83 (7.01–44.84) 18.71 (10.45–44.10) 12.83 (7.01–23.52)	*p* = 0.193 *	0.150
FFSTM (kg)—median (range) Females Males	41.71 (34.38–62.72) 38.37 (34.38–50.73) 54.26 (47.84–62.72)	*p* < 0.001 *	46.96 (32.92–66.46) 41.43 (32.92–53.38) 57.30 (46.96–66.46)	*p* < 0.001 *	0.413
SI (kg/m^2^) Females Males	6.57 (5.43–8.78) 5.86 (5.43–7.41) 7.92 (7.39–8.78)	*p* < 0.001 *	8.11 (5.62–9.41) 6.56 (5.62–8.27) 8.62 (7.76–9.41)	*p* < 0.001 *	**0.038**
Brain volume measures NBV (cm^3^)—mean ± SD GMV (cm^3^)—mean ± SD WMV (cm^3^)—mean ± SD T2LV (cm^3^)—median (range)	1520 ± 109 798 ± 71.9 722 ± 47.1 2.63 (0.21–24.8)	NA	NA	NA	NA
GSH (μM)—mean ± SD GSSG (μM)—mean ± SD	1127 ± 207 3.23 ± 1.41	NA	NA	NA	NA

* Comparison between men and women within the groups. Statistically significant *p* values are reported in bold. NA: not applicable.

## Data Availability

Access to research data is restricted by the authors for privacy reasons; data sharing for motivated reasons can be requested from the corresponding author.

## Supplementary Materials

The following supporting information can be downloaded at: https://www.mdpi.com/article/10.3390/jcm15135241/s1, Table S1. Association of body composition and MRI measures in pwMS that did not reach statistical significance; Table S2. Differences in median BC measures between patients with high or low levels of pSTAT; Figure S1. Correlation between serum NfL levels and BMI (A), FM (B), FFSTM (C) and SI (D).

## Abbreviations

The following abbreviations are used in this manuscript:

BC	Body Composition
BMI	Body Mass Index
CNS	Central Nervous System
DIR	Double Inversion Recovery
DMDs	Disease-Modifying Drugs
DXA	Dual-Energy X-ray Absorptiometry
EDSS	Expanded Disability Status Scale
FM	Fat Mass
FFSTM	Free Soft Tissue Mass
FLAIR	Fluid-Attenuated Inversion Recovery
Gd+	Gadolinium-Enhancing (Lesions)
GSH	Gluthatione
GSSG	Gluthatione Disulphide
GMV	Gray Matter Volume
HC	Healthy Controls
HPLC	High-Performance Liquid Chromatography
ITK-SNAP	Image ToolKit-Semi-automatic Nonlinear Atlas-based Processing
MRI	Magnetic Resonance Imaging
MS	Multiple Sclerosis
NEM	N-ethylmaleimide
NBV	Normalized Brain Volume
NfL	Neurofilament Light Chain
PPMS	Primary Progressive Multiple Sclerosis
pSTAT1	Phosphorylated STAT1
pwMS	Patients with MS
RRMS	Relapsing–Remitting Multiple Sclerosis
SD	Standard Deviation
SBG	Streptavidin-*β*-Galactosidase
SI	Sarcopenia Index
SIENAX	Structural Image Evaluation, Normalized, Voxel-based–FSL Tool for Volumetrics
SIMOA	Single Molecule Array
T2LV	T2 Lesion Volume
TCA	Trichloroacetic Acid
WMV	White Matter Volume
