# Supplementary material for: Body Composition Analysis in Young Patients with Recent Diagnosis of Multiple Sclerosis: An Exploratory Study
Source: J Clin Med. 2026 Jul 4;15(13):5241. doi: 10.3390/jcm15135241 (PMC13362906; doi:10.3390/jcm15135241)

Supplementary Table S1. Association of body composition and MRI measures in pwMS that did not reach statistical significance

BMI – median (range)<br>Gd+ lesions<br>No Gd+ lesions	 26.77 (25.73-27.80)<br>24.02 (19.31-36.06)	p=0.176
FM (kg) – median (range)<br>Gd+ lesions<br>No Gd+ lesions	 26.63 (23.16-30.11)<br>19.41 (10.42-44.84)	p=0.176
FFSTM (kg) – median (range)<br>Gd+ lesions<br>No Gd+ lesions	 47.22 (40.18-54.26)<br>41.71 (34.38-62.72)	p=0.618
SI (kg/m²) – median (range)<br>Gd+ lesions<br>No Gd+ lesions	 7.01 (5.91-8.12)<br>6.57 (5.43-8.78)	p=0.618
NBV – mean±SD<br>FFSTM < 41.71 kg<br>FFSTM > 41.71 kg	 1565.01±114.91<br>1456.49±67.28	p=0.09
GMV – mean±SD<br>FFSTM < 41.71 kg<br>FFSTM > 41.71 kg	 822.15±83.50<br>764.29±36.42	p=0.181
WMV – mean±SD<br>FFSTM < 41.71 kg<br>FFSTM > 41.71 kg	 742.86±46.20<br>692.21±32.12	p=0.062

Supplementary Table S2. Differences in median BC measures between patients with high or low levels of pSTAT

BMI – median (range)<br>Low pSTAT<br>High pSTAT	 25.62 (21.43-28.58)<br>22.92 (19.31-36.06)	p=0.377
FM (kg) – median (range)<br>Low pSTAT<br>High pSTAT	 21.04 (10.42-30.11)<br>17.31 (12.17-44.84)	p=0.510
FFSTM (kg) – median (range)<br>Low pSTAT<br>High pSTAT	 47.84 (34.38-62.72)<br>39.06 (37.64-52.75)	p=0.510
SI (kg/m²) – median (range)<br>Low pSTAT<br>High pSTAT	 7.39 (5.43-8.78)<br>6.30 (5.61-7.70)	p=0.583

Supplementary Figure S1. Correlation between serum NfL levels and BMI (A), FM (B), FFSTM (C) and SI (D)

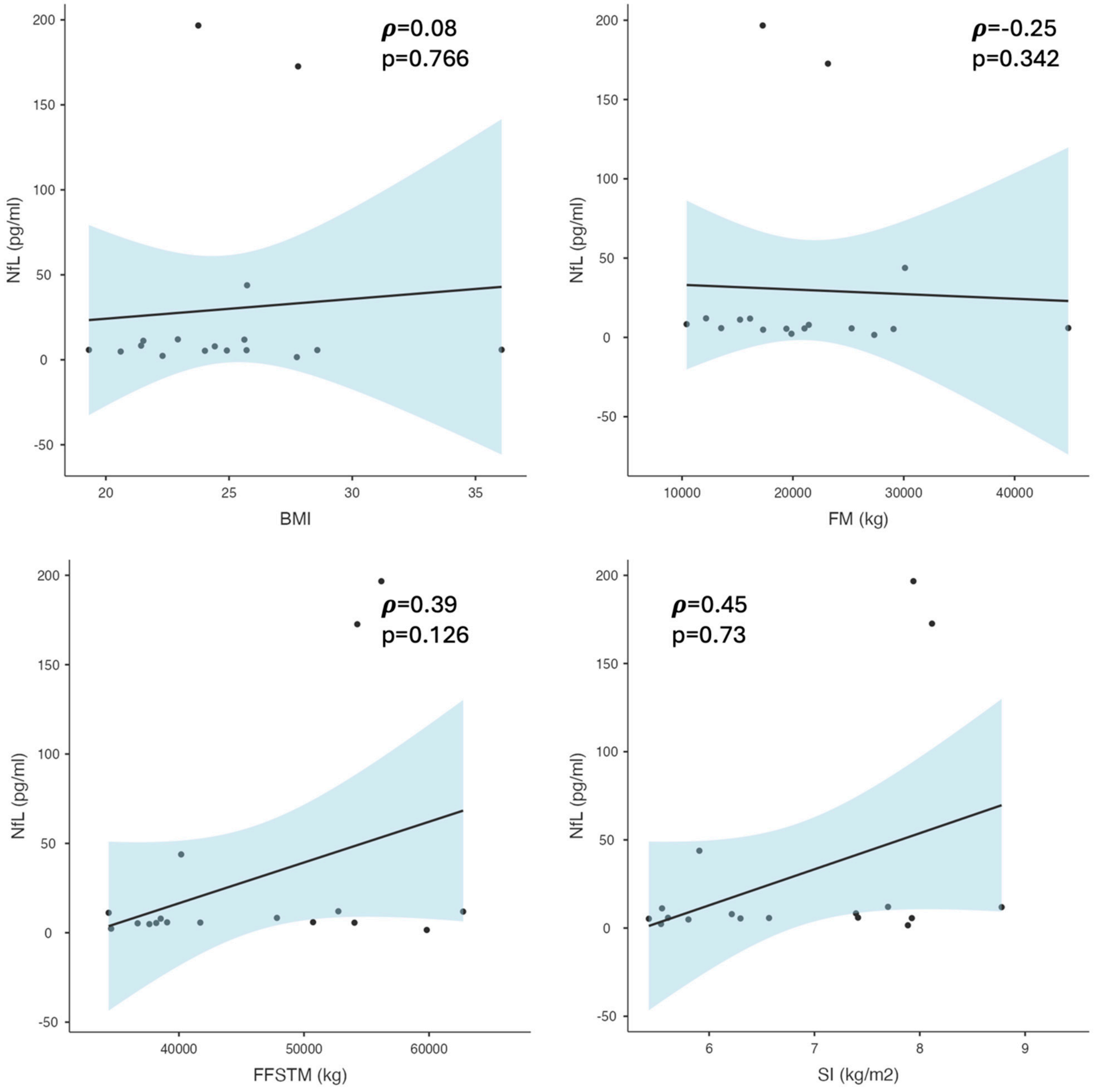

Supplement: Supplementary file 1 [file jcm-15-05241-s001.zip › jcm-4237471-supplementary.pdf]
